# Red Grape Pomace Rich in Polyphenols Diet Increases the Antioxidant Status in Key Organs—Kidneys, Liver, and Spleen of Piglets

**DOI:** 10.3390/ani9040149

**Published:** 2019-04-05

**Authors:** Veronica Sanda Chedea, Laurentiu Mihai Palade, Rodica Stefania Pelmus, Catalin Dragomir, Ionelia Taranu

**Affiliations:** Laboratory of Animal Biology, National Research and Development Institute for Biology and Animal Nutrition, Baloteşti (INCDBNA-IBNA), Calea Bucuresti nr. 1, Balotesti, 077015 Ilfov, Romania; palade_laurentiu_mihai@yahoo.com (L.M.P.); pelmus_rodica_stefania@yahoo.com (R.S.P.); catalin.dragomir@ibna.ro (C.D.); ionelia.taranu@ibna.ro (I.T.)

**Keywords:** grape pomace, total polyphenols, antioxidant status, piglets, blood biochemical parameters

## Abstract

**Simple Summary:**

Grape pomace (GP) is a residue generated by the wine industry that is produced in large quantities. This work aims to describe the beneficial effects replacing 5% of the corn in the diet of piglets with grape pomace. GP, besides other components, contains some important bioactive compounds called polyphenols. The study shows that the polyphenols from a 5% grape pomace diet are absorbed into the blood of piglets that receive it. These bioactive molecules have a beneficial action on the health status of the animals and also increase the antioxidant activity in the liver, spleen, and kidneys, which are key organs in the metabolism of the nutrients.

**Abstract:**

The aim of this work was to evaluate the influence of a diet containing 5% dried GP on the antioxidant status (total antioxidant status (TAS), antioxidant enzyme activity (catalase-CAT, superoxide dismutase-SOD, and gluthatione peroxidase-GPx), and lipid peroxidation) on the key organs of the liver, kidneys, and spleen in relation to health status as indicated by blood biochemical parameters and total polyphenol content in the blood, organs (liver, spleen, kidney, mesenteric lymph nodes, heart, and brain) and *Longissimus dorsi* muscle in piglets. The GP diet results in a significant increase of TAS in the liver, spleen, and kidneys, with increased CAT activity in the spleen and kidneys, increased SOD activity in the liver, kidneys, and spleen, and increased GPx activity in the kidneys, as well as a decrease in lipid peroxidation in the liver and kidneys. The GP included in the piglets’ feed contained polyphenols that showed antioxidant activity and were absorbed in the plasma, contributing to maintaining the good health of the animals. The inclusion of 5% GP inclusion in the diets of piglets is beneficial for overall normal blood constituent metabolism and helps to maintain piglet health by increasing the polyphenol content in blood plasma and antioxidant activity in the liver, spleen, and kidneys.

## 1. Introduction

Winery waste, which includes grape stalks, vine shoots, grape pomace, grape seed, yeast lees, etc. [1] forms approximately 20% of the mass of grapes [2]. Grape pomace (GP) consists mainly of skin residues, broken cells with pulp remains, stalks, and seeds [3]. It has been described that GP contains a great number of anthocyanins, catechins, flavonols, alcohols, and stilbenes [3]. Among the biological properties of GP, the best known and most widely studied is the antioxidant effect, which has been the focus of a large number of investigations, mostly of a clinical and nutritional nature [3]. 

The recent interest in natural biologically active polyphenols from GP has also grown in the field of animal nutrition, particularly with respect to their inclusion in the animal diet [4]. This interest is also associated with the possibility to mitigate the problems that arise from the decomposition of such waste in the environment [1]. In this context, using winery industry byproducts in animal feed would be an advantage. GP has been included in the feed of chickens [5], pigs [6], rabbits [7], cows [8,9], and sheep [10]. Worldwide concern about the negative effects of antibiotics led to their use being banned as growth promoters in the European Union on January 1, 2006. Bioactive compounds of grape pomace like polyphenols, unsaturated fatty acids, fiber, minerals, etc. have been considered beneficial in the prevention of inflammation and in antioxidant/antimicrobial processes, thus representing a solution in the form of replacement of antibiotics by bioactive phytochemicals.

In pigs, studies with grape by-products have been focused on bettering animal health and production, with subsequent improvements in the quality of the meat and digestibility. For example, in finishing pigs the fermented GP included in the diet at the level of 30 g/kg improved the growth performance and nutrient digestibility and altered the fatty acid pattern (total polyunsaturated fatty acids-PUFA and polyunsaturated fatty acids/saturated fatty acids-PUFA/SFA ratio) in the subcutaneous fat, as well as some attributes of pork meat [11]. Also in finishing pigs, Cho et al. (2012) investigated the effect of high moisture and fiber content of apple and grape meal fermented by *Lactobacillus reuteri* (100–200 g/kg) on proximate composition and amino acid digestibility, feces and urine excretion, fecal microbial, nitrogen balance, and the occurrence of volatile fatty acids [12]. They found that this feeding system increased the number of beneficial bacteria and decreased volatile fatty acids emission in feces [12]. 

Feeding a grape seed and grape marc extract (1%) as a dietary supplement in pigs might provide a useful dietary strategy to inhibit the inflammation in the gut that frequently occurs in pigs [6] during the weaning period. The dietary grape seed and grape marc extract improved the gain:feed ratio and decreased inflammation in weaning pigs without effects on the antioxidant system and vitamin E status [6,13]. An optimized polyphenol mixture extracted from apples, grape seeds, green tea, and olive leaves improved antioxidant status, including the plasma antioxidant activity of post-weaning piglets, and counteracted some of the negative effects that occur when piglets were challenged with *Escherichia coli* [14,15]. 

The beneficial actions of polyphenols in animals are largely dependent on their bioavailability at the target tissue [16], cellular distribution, and metabolism after absorption. In this context, there is a high interest in investigating the bioavailability and bioactivity of these components and their role in the modulation of redox status in vivo [17,18,19]. For instance, in piglets Kafantaris et al. (2018) investigated the effects of feed supplemented with GP on piglet productivity, redox status, microbiota, and meat quality, following the hypothesis that there might be a potential beneficial effect of feed supplemented with GP on animal health through an enhancement of various antioxidant mechanisms in piglets’ blood and tissues [18]. Moreover, in piglets, Gerasopoulos et al. (2015) demonstrated that feed supplemented with polyphenolic byproduct from olive mill wastewater processing improved the redox status in the blood and tissues of piglets [19].

In this direction, and because the studies on the effect of grape by-products in piglets are limited, the aim of this work was to evaluate the influence of a diet containing 5% dried GP on general health status and polyphenol bioavailability and storage in different organs of growing piglets, as well as the effect of dietary polyphenol on antioxidant status (catalase (CAT), superoxide dismutase (SOD), and glutathione peroxidase (GPx) activity, total antioxidant status (TAS), and 1,1-Diphenyl-2-picrylhydrazyl (DPPH) antiradical activity and lipid peroxidation (by assessing the thiobarbituric acid reactive substances-TBARS levels) on different key organs.

## 2. Materials and Methods 

### 2.1. Animals and Diets 

For this study, a total number of 20 crossbred TOPIG hybrid ((Landrace × Large White) × (Duroc × Pietrain)) pigs with an average body weight of 10.70 ± 0.8 kg were allocated to two experimental groups (10 pigs/group) [20]. The animals, individually identified by ear tags, were housed in pens (5 pigs/pen) and fed isoenergetic and isoproteic experimental diets containing 5% grape pomace (experimental group, GP+) or 0% GP (control group, GP−) for 36 days (Table 1) [20]. 

The dry matter (DM), crude protein (CP), fat (EE), crude fiber (CF) and ash of basal and experimental diets, as well as of grape pomace were determined according to ISO methods (ASRO-SR EN ISO, 2010). The following values resulted: 88.87% (GP−), 88.90% (GP+) and 87.63% (GP) for DM; 18.53% (GP−), 18.71% (GP+) and 10.32% (GP) for CP; 2.18% (GP−), 2.70% (GP+) and 5.14 % (GP) for EE; 4.22% (GP−), 5.36% (GP+) and 25.01% (GP) for CF; 6.12% (GP−), 5.19% (GP+) and 5.75% (GP) for ash.

The grape pomace was provided by a local producer and derived from Valea Calugareasca, a Romanian winery [20]. The pomace consisting of stems, skins and seeds, was dried in a heated airflow. GP raw material was milled to a particle size of less than 6 mm in a Cyclone Mill—MC5 (Tecator, Höganäs, Sweden) and incorporated, 5%, into the conventional compound feed. (Table 1) [20]. In respect of the beneficial health implications, we considered the dose of 5% GP incorporation in the regular feed of the piglets as being safe from the animals’ nutritional point of view for normal growth and without lowering the productive parameters. The dietary formulations were in agreement with National Research Council (NRC) (2012) nutrient recommendations for the physiological state of piglets. 

Pigs had free access to the water and assigned diet during the 36-d experimental period. The body weight was recorded at day 0 and at day 36 for each animal and feed intake (ADFI); the feed:gain (F:G) ratio was recorded daily per pen as already presented [20]. 

The animals were cared for in accordance with the Romanian Law 43/2014 for handling and protection of animals used for experimental purposes and the EU Council Directive 98/58/EC concerning the protection of farmed animals [20]. The study protocol was approved by the Ethical Committee of the National Research-Development Institute for Animal Nutrition and Biology, Balotesti, Romania (Ethical Committee no. 52/2014, approval no. 3220/27.05.2016) [20]. All animals remained healthy during the experimental period and no veterinary drugs were used. All efforts were made to minimize suffering at slaughter [20]. 

### 2.2. Sample Collection

After 15 days, and at the end of the experiment, blood samples were aseptically collected from 2 × 10 piglets from the jugular vein. The blood samples were aseptically collected into 9-mLVacutainer tubes containing 14.3 U/mL of lithium heparin (Vacutest®, Arzergrande, Italy), then separated by centrifugation at 4914 rpm for 25 min at 4 °C. Plasma, the red cells sediment and whole blood were stored at −80 °C until assayed (biochemistry, UV-Vis spectroscopy and total polyphenols content-TPC). 

At the end of the experiment (36 days) animals were slaughtered, and samples were collected from the following organs: Liver, spleen, kidney, mesenteric lymph nodes, heart and brain, and from *Longissimus dorsi* muscle. The samples were stored at −80 °C until analyzed (TPC and antioxidant activity). The frozen organ samples were milled in liquid nitrogen (IKA works, 2900000 A11 basic Analytical Mill).

### 2.3. Extraction of Polyphenols and Determination of the Total Phenolic Content (TPC) from Feed

The polyphenols from the compound feed and from the feed ingredients (corn grains, wheat grains, sunflower meal, soybean meal), including the dried GP, were extracted (sample: solvent ratio of 1:7 w/v) in acetone 80% and methanol (100%) for 24 hours at 37 °C, under continuous shaking. The mixture was then vortexed vigorously for 3 minutes followed by centrifugation for 15 min at 14,800 rpm at room temperature. The supernatants representing the methanolic and acetone extracts were collected and kept at −20 °C until further analyses. The total polyphenolic content was determined with the Folin-Ciocalteu method, as previously described [9].

### 2.4. Extraction of polyphenols and Determination of the Total Phenolic Content (TPC) from Plasma and Organs (Liver, Spleen, Kidney, Mesenteric Lymph Nodes, Heart and Brain) and Longissimus Dorsi Muscle

A methanolic (100%) extraction of polyphenols (ratio, sample: solvent being 1:4 v/v) was done for the samples of piglet plasma, cell sediment and whole blood collected after 15 days, and at the end of the experiment. The mixture was then vortexed vigorously for 3 min followed by centrifugation for 15 min at 14,800 rpm at room temperature. The supernatants representing the methanolic extracts were collected and stored at −20 °C until further analysed.

The polyphenols from organs were extracted with methanol 100%. One gram of frozen sample powder was mixed with 10 ml MetOH and crushed with an Ultra Turrax (Jahnke and Kunkel, IKA, Staufen, Germany) [22]. The mixture was treated for 30 minutes in an ultrasonic water bath at 4 °C and centrifuged for 10 minutes. The supernatant was kept at −20 °C until further analyses. TPC was determined using Folin–Ciocalteu method [23] adapted to microscale [24] as follows: 40 μL of plasma, red cells sediment, whole blood, muscle and organs extracts or 20 μL of feed and ingredients extracts, were mixed with 1560 μL (in case of plasma, red cells sediment, whole blood, muscle and organs extracts) or 1580 μL (in case of feed) of distilled water, plus 100 μL of Folin–Ciocalteu reagent and vigorously stirred. After exactly 1 min, 300 μL of aqueous sodium carbonate 20% was added, and the mixture was vigorously stirred again and allowed to stand at room temperature in the dark, for 90 min. Absorbance was then read at 750 nm (on a UV-visible diode array spectrophotometer Specord 250, Analytik Jena, Jena, Germany), and TPC was calculated from a calibration curve, using gallic acid as standard. The results were expressed as mg gallic acid equivalents (mg GAE)/100g raw material (r.m.) for feed and its components or mg GAE /100g tissue in case of organs and muscle or mg GAE/L in case of plasma, red cells sediment and whole blood analysis.

### 2.5. Evaluation of the Antioxidant Activity Using the DPPH Method (AAR) in Organs (Liver, Spleen, Kidney, Mesenteric Lymph Nodes, Heart and Brain) and LONGISSIMUS DORSI MUSCLE 

1,1-Diphenyl-2-picrylhydrazyl (DPPH) is a stable free radical and has been commonly used to screen phenolic compounds containing high free radical scavenging ability [25]. AAR was determined using the DPPH test according to Arnous et al. (2001) with slight modifications [24]. An aliquot of 20 μL of organ (liver, spleen, kidney, mesenteric lymph nodes, heart and brain) and L. dorsi muscle methanolic extract was added to 980 μL of DPPH solution (60 μM in methanol), vortexed, and the absorbance was read at t = 0 (A 515(0)) and t = 30 (A 515(30)) min using a Specord 250 (Analytik Jena, Jena, Germany) array spectrophotometer. The AAR was determined as follows: %∆A515 (μM Trolox Equivalents TE) = 0.0921× AAR + 2.3146 as determined from linear regression, after plotting %∆A515 of known solutions of Trolox against concentration (85–800 μM, R2 = 0.995) where:

%∆A515 = [(A 515(0) − A 515(30))/A 515(0)] × 100.(1)

### 2.6. Preparation of Tissue Lysates 

Frozen tissue (liver, spleen and kidneys) samples (200 mg) were disrupted and homogenized using Ultra-Turrax homogenizer (IKA-Werke GmbH and Co. KG, Staufen, Germany). 50 mM potassium phosphate (pH 7.0, containing 1 mM EDTA per gram tissue) for CAT, 20 mM HEPES buffer (pH 7.2, containing 1 mM EGTA, 210 mM mannitol and 70 mM sucrose per gram tissue) for SOD, 50 mM Tis-HCl (pH 7.5, containing 5 mM EDTA and 1 mM DTT per gram tissue) for GPx and 1 × PBS (EDTA free) for TAS and TBARS were added. The homogenates were kept 30 min on ice, and then centrifuged at 10,000 g at 4 °C for 10 min. If not assayed immediately (by the TBARS-MDA, TAS, CAT activity, SOD activity GPx activity assays from liver, spleen and kidneys), the supernatants (tissue lysates) were frozen at −80 °C (stable for 1 month).

### 2.7. Measurement of Tissue Lipid Peroxidation—TBARS-MDA (Liver, Spleen and Kidneys)

Lipid peroxidation was analyzed as previously described by Ohkawa et al. [26]. The mixture of reaction contained 10 µL of the sample (appropriately diluted tissue lysate at a concentration of 1000 µg protein/ml), 240 µL of deionized water, 25 µL of 0.5 N HCl and 250 µL thiobarbituric acid (TBA). The mixture was incubated at 95 °C for 15 minutes and the reaction was stopped by an immediate transfer of each tube to ice. Subsequently, the reaction mixture was added to a microplate well and measured in fluorescence mode (exc. 515 nm; em. 548 nm). The results were expressed as nmol MDA/g tissue, using 1,1,3,3, -tetramethoxypropane (TMP) as standard.

### 2.8. Measurement of Total Antioxidant Status (TAS) (Liver, Spleen and Kidneys)

The antioxidant capacity of the tissue samples was measured with the TEAC (Trolox equivalent antioxidant capacity) assay using ABTS as radical cation (2,2’-azinobis-(3-ethylbenzothiazoline-6-sulphonic acid), a method adapted from Miller et al. (1993) [27]. Briefly, 30 µL ABTS, 10 µL metmyoglobin and 127 µL of buffer (of which 10 µL were replaced with the appropriately diluted sample which was being investigated: 1:40 for liver, 1:30 for spleen, 1:100 for kidneys) were mixed and then incubated at 37 °C for 3 min. The absorbance was measured at 732 nm. The reaction was then initiated by the addition of 33 µL of hydrogen peroxide. Spectra were recorded again at 732 nm. The results were expressed as mmol/L Trolox equivalents (plasma) and µmol TE/g of tissue (organ tissue).

### 2.9. Measurement of Tissue Catalase (CAT), Superoxide Dismutase (SOD) and Glutathione Peroxidase (GPx) Activity (Liver, Spleen and Kidneys)

Catalase activity was measured using Catalase Assay Kit (Cayman Chemical) according to the manufacturer’s instructions. Briefly, 100 µL of diluted Assay Buffer, 30 µL of methanol, and 20 µL of the sample (appropriately diluted tissue lysate: 1:2000 for liver, 1:100 for spleen, 1:500 for kidneys) were mixed with 20 µL of hydrogen peroxide in each well of a 96 wells plate; the plate was then covered and incubated on a shaker for 20 minutes at room temperature. 30 µL of potassium hydroxide was added were used to stop the reaction. After a 10-minute incubation on the shaker with Catalase Purpald (30 µL per well), the solution was finally incubated with potassium periodate (5 minutes, 10 µL per well). Absorbance was read at 540 nm using a microplate reader (Tecan Infinite M200). Results (CAT activity) were expressed as µmol/min/g tissue. One unit was defined as the amount of enzyme that will cause the formation of 1.0 nmol of formaldehyde per minute at 25 °C.

Superoxide dismutase activity was measured by using a SOD Cayman Assay Kit according to the manufacturer’s instructions. Briefly, 200 µL of diluted Radical Detector and 10 µL of the sample (appropriately diluted tissue lysate: 1:100 for liver, 1:200 for kidneys, 1:100 for spleen) were mixed and the reaction was initiated by adding 20 µL of diluted Xanthine Oxidase to each well. Then, the plate was covered and incubated on a shaker for 30 minutes at room temperature. Absorbance was read at 440–460 nm with a microplate reader (Tecan Infinite M200). Results (SOD activity) were expressed as U/g tissue. One unit was defined as the amount of enzyme needed to exhibit 50% dismutation of the superoxide radical. SOD activity is standardized using the cytochrome c and xanthine oxidase coupled assay.

A Glutathione Peroxidase Assay Kit manufactured by Cayman Chemical was used. Briefly, 100 µL of final Assay Buffer, 50 µL of GPx Co-Substrate Mixture, and 20 µL of the sample (appropriately diluted tissue lysate: 1:100 for liver, 1:30 for spleen and 1:100 kidneys) were added to each well of a 96 well plate (Greiner ®). The reaction was initiated by the addition of 20 µL of GPx Cumene Hydroperoxide to each well and the plate was carefully mixed for a few seconds. The absorbance was read every minute at 340 nm with a plate reader (Tecan Infinite M200). Results (GPx activity) were expressed as µmol/min/g tissue. One unit was defined as the amount of enzyme that will cause the oxidation of 1.0 nmol of NADPH to NADP+ per minute at 25 °C.

### 2.10. Measurement of UV-VIS Spectra from Liver, Spleen and Kidneys Samples

The spectra for liver, spleen and kidneys samples, as methanolic extracts, were recorded at room temperature using a spectrophotometer (Specord 250, Analytik Jena, Jena, Germany) in the UV–Vis range 250-1050 nm [28].

After registering the UV-Vis spectra of all samples (L-, L+, S-, S+, K- and K+) the control spectra, L-, S- and K- respectively, were subtracted from each L+, S+ and K+ spectrum, the λmax of the remaining spectra was determined, using the software of the spectrophotometer. These subtractions were overlayed using the Overlay function of the apparatus [28].

### 2.11. Measurement of Plasma Biochemical Parameters

Concentration of plasma biomarkers, glucose, total cholesterol, triglycerides, total protein, albumin, bilirubin, creatinine, urea, P, Ca, Mg, Fe and the activity of alkaline phosphatase (ALP), alanine transaminase (ALT), aspartate transaminase (AST) and gamma-glutamyl transferase (GGT) were determined on an automatic BS-130 Chemistry analyzer (Bio-Medical Electronics Co., LTD, China), according to the manufacturer instructions, from plasma of blood collected, as described in “sample preparation” section, after 15 days of treatment and at the end of the experiment. 

### 2.12. Statistical Analysis 

The results were presented as mean values ± standard errors of the mean (SEM) from at least three independent measurements. Each pig was considered an experimental unit.

For the plasma biochemical parameters, ANOVA GLM procedure of Minitab 16 software, followed by Tuckey test, was used for assessing the differences between groups, within an experimental design having as fixed factors the diet (D), day of sampling (T) and their interaction-diet and day of sampling (D × T). 

All the other experimental data were analyzed with the program performing one-way analysis of variance (ANOVA), followed by a Fisher protected least significant difference (PSLD) test. *p*- values lower than 0.05 were considered significant while *p* values between 0.05 and 0.1 were considered as tendencies.

## 3. Results

### 3.1. Total Polyphenols Content (TPC) and Antioxidant Activity (AAR) in Feed Compound and Its Components

Two solvents, methanol and acetone, were used for polyphenol extraction from the experimental diets (GP−and GP+) and from their ingredients (corn grains, wheat grains, sunflower meal, soybean meal and dried grape pomace, Table 2). 

The highest number of polyphenols was extracted with acetone (Table 2). The highest TPC has been found in the sunflower meal, followed by dried GP, while the lowest TPC was determined in corn and wheat (Table 2). As expected, the diet containing GP (GP+), had a higher level of polyphenols than the control (GP−) diet (Table 2). When methanol was the extraction solvent, sunflower meal also had the highest TPC content, but lower by almost 50% compared to acetone, followed by dried GP, soybean meal, wheat grains and corn grains (Table 2). Also, in the case of methanol extraction, the diet containing GP (GP+), had a higher TPC than the control (GP) diet, similarly with the acetone extraction (Table 2), but lower by almost 10%. 

When acetone was the extraction solvent, the antioxidant activity reached the highest level for GP+ diet and sunflower meal, with similar AAR values. Soybean meal followed and then the dried grape pomace and GP−diet, with the lowest activity recorded for cereals, wheat and corn grains (Table 2). In the case of methanol extraction, as for acetone, the sunflower meal had the highest activity, followed by the dried grape pomace, soybean meal, GP+ diet, wheat grains, corn grains and GP-diet (Table 2). For DPPH test, all values were statistically different (on the column, as well as on row, Table 2), excepting the sunflower meal for which no significant difference between the acetone and methanol extracts was found. 

Using liquid chromatography coupled with mass spectroscopy, we determined earlier that GP has the following polyphenols in its composition: A procyanidin trimer (possibly C2) found in the highest amount (16.54 mg Catechin Equivalents/100 g), followed by a procyanidin dimer and equally by gallic acid-glucoside, gallic acid, and a procyanidin trimer C1 [20]. Malvidin 3-O-(6″-coumaroyl-glucoside) was found in the lowest concentration in GP [20].

### 3.2. Effect of Dietary GP on Performance

As showed by Chedea et al., [20] there were no significant differences in ADG, and F:G among groups, although a slight increase in total ADG (613.89 g vs. 570.99 g) and F:G (2.10 vs 1.98) was noticed in the piglets fed the GP+ diet. However, a significant increase for ADFI was noticed when GP was administrated (1255.14 g vs. 1097.33 g) [20].

### 3.3. Effect of Dietary GP on Health Status (Plasma Biochemical Parameters)

There was a significant effect of GP+ time (T) administration and also of its interaction with diet (D × T) on lowering triglyceride concentration (T, *p* = 0.04, and D × T, *p* = 0.046) and increasing albumin in plasma of the pigs receiving GP+ diet for 36 days (Table 3). Phosphorus and ALP increased and calcium decreased in time (T) independently of the diet (Table 3). Time (*p* = 0.069) and the interaction between D and T (*p* = 0.068) had a statistically marginal effect on total cholesterol (Table 3). 

### 3.4. Total Polyphenols Content (TPC) in Blood and Its Fractions

Table 4 shows blood TPC and its fractions—cells sediment and plasma- collected after 15 days of GP feeding, and at the end of the experiment (36 days). The GP+ diet, and the time of administration influenced significantly the plasma TPC. Indeed, at the end of the experiment (d 36) plasma had a higher TPC compared with the control, and with GP+ plasma collected after 15 days (Table 4). GP+ diet had a tendency to increase the presence of polyphenols in the cell sediment fraction. In case of total blood, the time, but not the diet, increased the TPC. 

### 3.5. Total Polyphenols Content (TPC) and Antiradical (DPPH) Activity (AAR) in Organs (Liver, Spleen, Kidney, Mesenteric Lymph Nodes, Heart and Brain) and LONGISSIMUS DORSI MUSCLE

TPC and AAR of organs (liver, spleen, kidney, mesenteric lymph nodes, heart and brain) and of Longissimus dorsi muscle measured at the end (36 d) of the experiment are presented in Table 5. GP+ diet determined a significant higher TPC in the kidney (*p* = 0.019), but had no effect on TPC level in the other organs. Irrespective of the diet, liver had the highest TPC concentration followed by the brain, kidney and spleen (almost equal), heart, lymph nodes and, finally, the Longissimus dorsi muscle with the lowest TPC (Table 5).

The highest AAR was registered in the liver and the lowest in heart, 7.64% of liver AAR (Table 5). The descending order of AAR for the other tissue samples was: Lymph nodes, spleen, brain, kidney and Longissimus dorsi muscle (Table 5).

### 3.6. Effect of GP Diet on Antioxidant Status in Liver, Spleen and Kidney

The antioxidant status-assessed by measuring the levels of TAS, antioxidant enzymes activity (CAT, SOD and GPx) and TBARS-MDA, was further determined in the liver, spleen and kidney, due to the importance of these organs for the metabolization, excretion and health-immune status of GP polyphenols and other bioactive compounds.

**Figure 1:**
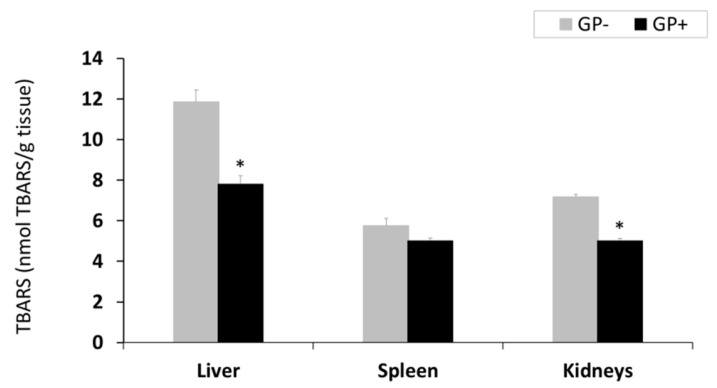
Lipid peroxidation (nmol TBARS/g tissue) as determined by the TBARS assay, in the liver, spleen and kidneys samples (n = 10); * = statistically significant when GP− was compared with GP+.

Lipid peroxidation (TBARS-MDA). TBARS-MDA concentration decreased in all analyzed organs derived from piglets fed GP+ diet being significantly only in the liver (−34%) and kidney (−30%, Figure 1). However, the TBARS level was higher in the liver compared to the other organs irrespective of the diet (Figure 1).

**Figure 2:**
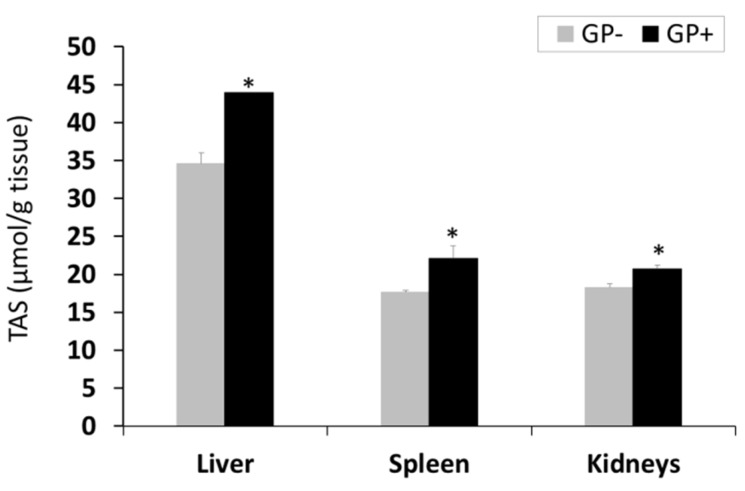
Total antioxidant status (µmol/g tissue) as determined by the total antioxidant status (TAS) assay, in the liver, spleen and kidneys samples (n = 10); * = statistically significant when GP− was compared with GP+.

Total antioxidant status (TAS). Assessment of TAS showed a higher antioxidant status in the liver compared to the spleen and kidneys; for all three organs, a significant (*p* < 0.05) increase was noticed in the case of GP+ diet compared to the control (43.94 μmol/g tissue GP+ vs 34.63 μmol/g tissue GP−for liver, +21%, 22.15 μmol/g tissue GP+ vs 17.68 μmol/g tissue GP−for spleen, +20% and 20.71 μmol/g tissue GP+ vs 18.3 μmol/g tissue GP−for kidneys +11%, (Figure 2). 

**Figure 3:**
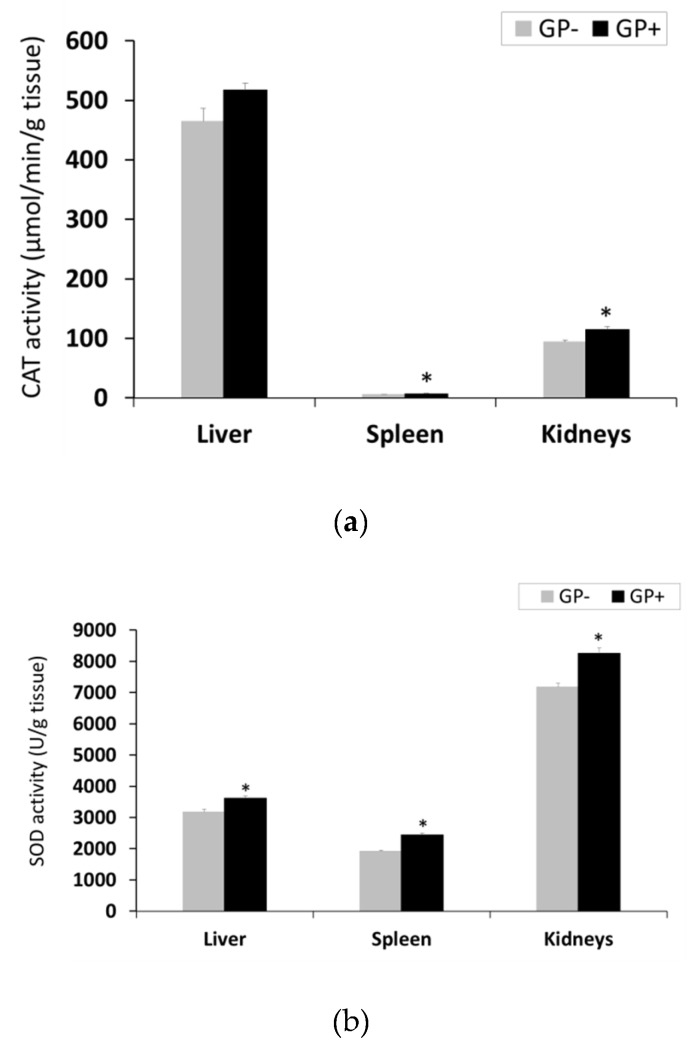

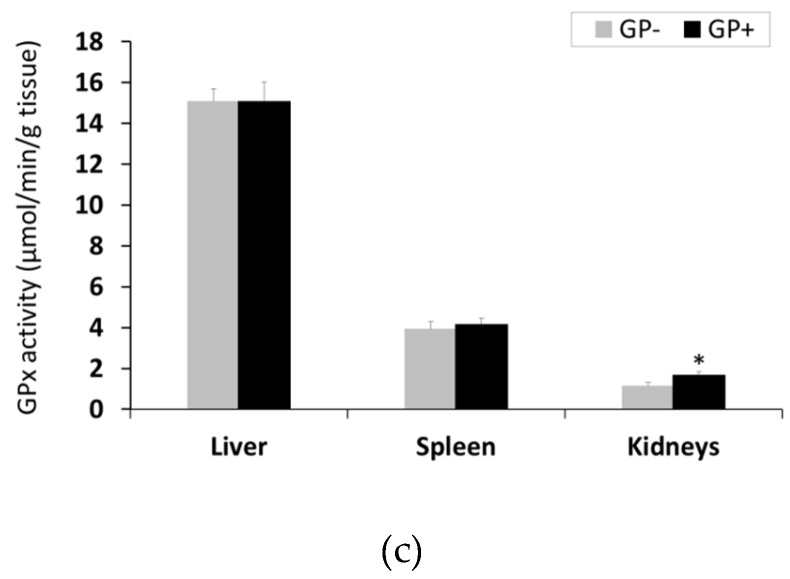
Antioxidant activity in the liver, spleen and kidneys samples (n = 10) as determined by: Catalase (CAT) activity (µmol/min/g tissue) (**a**), superoxide dismutase (SOD) activity (U/g tissue) (**b**), glutathione peroxidase (GPx) activity (µmol/min/g tissue) (**c**); * = statistically significant when GP− was compared with GP+.

Antioxidant enzymes activity. GP diet led to a significant increase of CAT activity in the spleen, by 13% and in kidneys, by 18%, with higher concentrations in the kidneys compared to the spleen (Figure 3A). A strong tendency of increasing CAT activity in the liver (+10%) (*p* = 0.055) was also observed (Figure 3A). Irrespective of the diet, SOD had higher activity in the kidneys followed by liver and spleen. With regard to GP+ diet, a significant increase in SOD activity in the kidney (+ 13%), in spleen (+21 %) and in the liver (+11%) was found (Figure 3B). GPx activity remained unchanged in the liver and spleen tissues, irrespective of the diet, but increased significantly in the kidneys of pigs fed GP+ diet (+33%, Figure 3C).

### 3.7. Qualitative Assessment of Polyphenols Absorption in Liver, Spleen and Kidney by UV-Vis Spectra Measurement

Table 6 shows all the absorption maxima registered for liver, spleen and kidney samples. 

**Figure 4:**
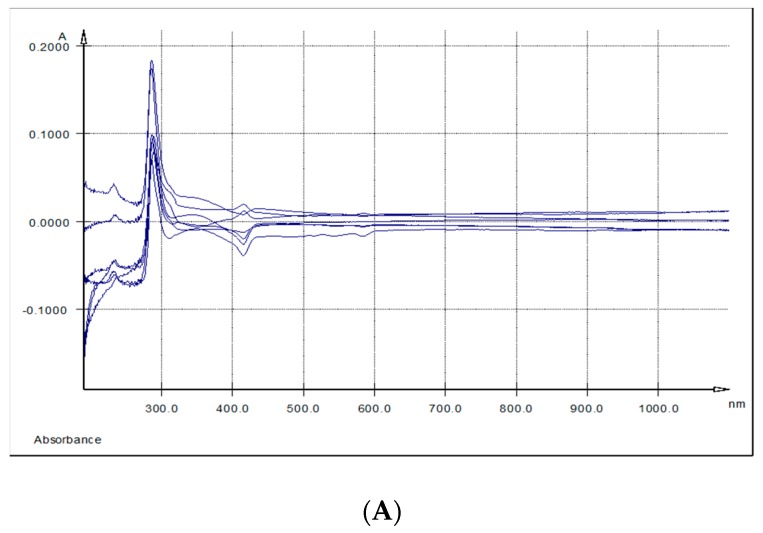

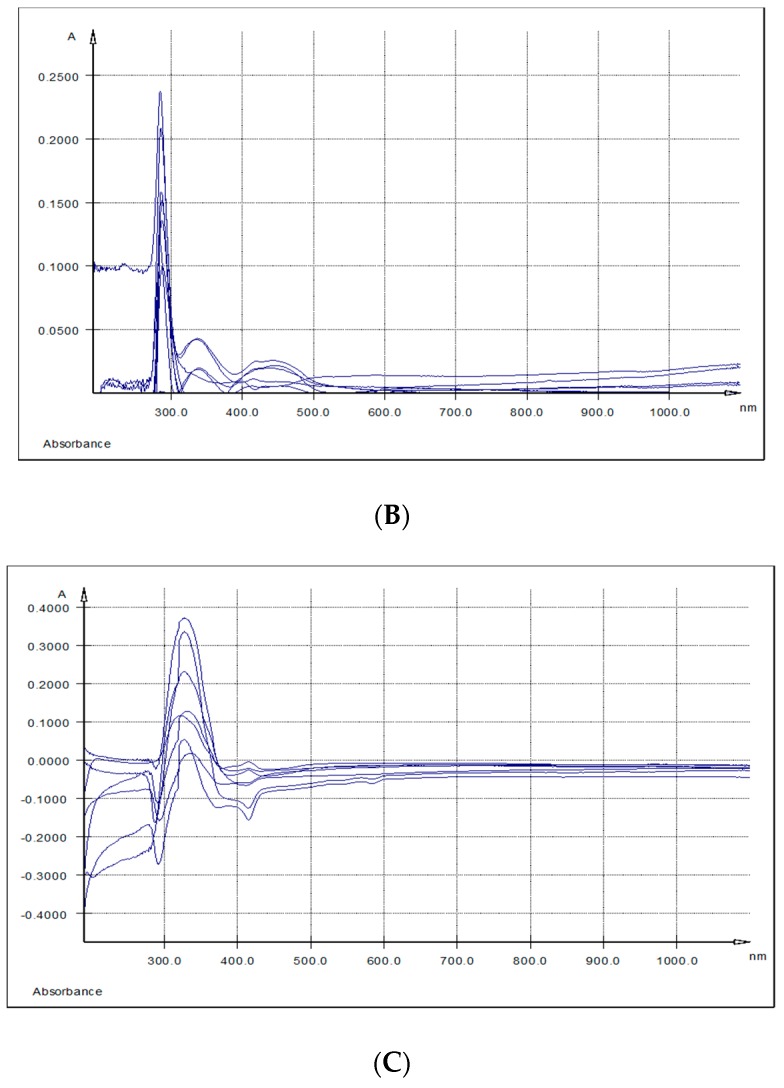
UV-Vis spectra: (**A**) Differences between spleen samples originating from the experimental (5% GP diet) and the control (no GP diet) groups; (**B**) differences between kidneys samples originating from the experimental (5% GP diet) and the control (no GP diet) groups; (**C**) differences between liver samples originating from the experimental (5% GP diet) and the control (no GP diet) groups.

The overlaid UV-Vis spectra of spleen and kidney respectively methanolic extracts, indicated three absorption bands. The first band (I) had a maximum of absorption at λ max = 284.6–289 nm, the second band (II) at λ max = 336 nm (kidney) and λ max = 344 nm (spleen) and the third band (band III) at λ max = 415 nm (spleen) and λ max = 433 nm (kidney) (Table 6 and Figure 4A,B). 

The UV-Vis spectra of liver samples reveal only two absorption bands: band II having the absorption maxima at λ max = 328 nm, the most intense one, band III at λ max = 430 nm (Table 6 and Figure 4C).

## 4. Discussion

High production volumes, environmental impact and nutritional content of the wine industry by-products make them an important subject for careful valorization, such as in animal feed [15]. In this study, weaned pigs were fed diets containing, or not, 5% dried grape pomace and its effect on the general health stat and antioxidant status of the piglets was monitored. In a previous study [20], we showed that feeding piglets diets containing or not 5% dried grape pomace, determined a significant increase in the average daily feed intake (ADFI) without significantly affecting the body weight in the case of GP diet. The analysis of plasma biochemical parameters, in this study, as general health status indicators, showed significant decrease in triglycerides for GP+ group after 36d of GP feed intake.

One potential disadvantage of grape pomace, fed in relatively high amounts, is that mineral absorption could be affected by the tannins present in grape products [29,30]. This was not an issue in this study, the plasma minerals not being decreased by the GP diet, and this may indicate the beneficial effect of feeding dried grape pomace as a matrix, as it is, and not as an extract. 

This result might be important if we take into consideration that pig is considered to be the most suitable non-primate animal model for human studies, due to its resemblances to the human situation, higher than in any other non-primate animal species [31]. These include similar development of the intestine in many features during fetal and postnatal development, the small intestine being relatively mature at birth for both species [32,33,34]. Fat digestion and absorption [32,33,34], as well as the digestive function of the newborn piglet and human infant have many similarities in terms of enzyme activity and digestive capacity [34]. Body composition is very similar in pigs and man at birth, 3 months, 3 years for pigs and 33 years for man) and both man and pigs are dependent on the dietary quality (for example, amino acids, digestible carbohydrates) [34]. The triglycerides decrease indicates the cardio-protective properties of dietary GP. In human, high levels of triglycerides are usually associated with coronary heart disease, pancreatitis and numerous genetic lipoprotein disorders (CVDs) [35]. The same triglycerides lowering effect, was registered when the diet of growing pigs was supplemented with dried olive leaves rich in polyphenols, such as oleuropein [36].

“In vivo” animal feeding trials [4,9], as well as, human clinical studies [37] describing the quantifications of antioxidant systemic levels, mainly involved plasma measurements [37]. In this study, for a better view of TPC distribution, we determined the TPC in three blood fractions: Plasma, cell sediment and whole blood. The plasma of the piglets fed the GP+ diet a longer period (36 days) had the highest number of polyphenols, a significant interaction between diet (D) and time (T) being observed (D × T, *p* < 0.026). The same situation was observed in the plasma of dairy cows fed a 15% GP diet, when the level of total polyphenols was determined [9]. 

The ability of blood cells to bind antioxidant polyphenols may be an important phenomenon with far-reaching *in vivo* consequences and so, it was concluded that it is paramount that antioxidants are to be measured in whole blood and not in plasma alone [38]. By contrast, the results of the present study concerning the TPC in the red cells sediment and in the whole blood of the piglets receiving GP+ diet did not show a significant presence of polyphenols when compared to the control (Table 3). 

Further, TPC distribution in organs (brain, heart, liver, kidney, spleen and lymph nodes) and in LD muscle was monitored, and from all the tested samples, only in the kidney (*p* = 0.019), GP+ diet determined a significant higher TPC storage. Serra et al. (2011) detected in kidneys too, a high number of polyphenol metabolites [39]. Differences in the tissue polyphenols may be due to the differences in the nature of the metabolites from different tissues and may be related to the specific uptake or elimination of some of the tissue metabolites or the intracellular metabolism [39]. In order to determine if these differences in the nature of the metabolites from studied tissues are present also in our case, we measured the UV-Vis spectra of the studied key organs, liver, spleen and kidneys, methanolic extracts. The modified spectrum, when compared to the GP extract, showed that the polyphenols were mainly structurally modified by metabolization, and that in this form they were absorbed in the liver, spleen and kidneys at λmax between 284.6 nm and 443.0 nm. This shift from 270 nm λmax of AGP shows oxidation following the ingestion. As in the case of *in vitro* experiments [28], the absorption maxima between 328 nm and 443 nm might be explained by and extensive oxidation at inter and intra molecular level between polyphenols and proteins, or other antioxidants and molecules, with the formation of *o*-quinones and dimers, possibly trimers, as shown by previous LC-MS results [20,40]. The analysis of polyphenols in different rat tissue samples, after the administration of a GP extract, showed intense metabolism in the liver [41] and monomethylated, dimethylated and trimethylated metabolites were reported for catechin and di- and tri-meric procyanidins [42]. 

Zhu et al., (2012) showed that weaning of piglets was also associated with the reduction of antioxidant mechanisms [43]. This hypothesis has been supported by the findings of Gerasopoulos et al (2015), since all the oxidative stress markers in the blood of the control group were improved post weaning [19]. Namely, markers showing antioxidant capacity (i.e., TAS, GSH and CAT) increased, while markers indicating oxidative damage (i.e., protein oxidation and lipid peroxidation) decreased in the control group, post weaning. Thus, it seems that oxidative stress is induced at weaning, and/or, that the antioxidant mechanisms did not develop adequately. Several studies have suggested the supplementation of feeds with antioxidants as a means of reducing the detrimental effects of the oxidative stress on animal health [19,43,44,45]. 

Polyphenols represent important nutrients brought by GP in diet and they are compounds with great antioxidant potential. It has been suggested that feeding diets rich in polyphenols could improve the antioxidant status of plasma and tissues [6]. For instance it is well documented that grape seed procyanidin extract can protect the functions of major organs by improving the antioxidant system, as well as prevent liver injury caused by carbon tetrachloride and ischemia/reperfusion [46]. However, the published literature regarding the polyphenols content and the antioxidant status of tissues when long term feeding a diet rich in polyphenols in piglets is inconclusive [6].

In the study herein, in the liver—the central site for nutrient metabolization and deposition—significant higher levels of TAS and AAR were recorded for the GP+ group compared to the control, even though there is no difference in TPC between the two groups. A direct correlation between TPC, TAS, AAR, and all the tested antioxidant enzymes, SOD, CAT and GPx, with levels higher than the control for the experimental variant, was found for kidneys. In the kidneys of piglets fed a 9% GP diet, Kafantaris et al (2018) also observed that total antioxidant capacity levels were significantly increased in the GP group compared to the control group [18]. Gerasopoulos et al., (2015) reported a significant increase of total antioxidant capacity in kidneys and spleen in piglets receiving 4% OMWW diet compared to the control group [19]. 4% of a polyphenolic byproduct from olive mill wastewater (OMWW) diet determined also an increase in the liver and kidneys CAT levels compared to the control group [19]. Di Giancamillo et al. [47] assessed both systemic and local responses to oxidative stress using verbascoside—a water-soluble derivate of phenylpropanoids, and a powerful antioxidant—to decrease the response to stress induced by a high supplementation of sunflower oil [47]. The results indicate that verbascoside supplementation in a high fat diet partially restores the antioxidant status of the liver, decreasing the oxidative stress by increasing the levels of SOD [47].

In our study, significant higher antioxidant actions of polyphenols were measured in the spleen for TAS, and also for SOD and CAT activity, while no correlation between TPC and the antioxidant parameters was found. A strong tendency of increasing CAT activity and significant SOD activity were also observed in the liver of piglets after 36 days of 5% GP diet. 

In liver, significant lower lipid peroxidation, expressed by the TBARS-MDA levels, were determined for the GP+ group compared to the control, and the same result was reported by Kafantaris et al. (2018) [18] in case of piglets fed 9% GP diet and by Gerasopoulos et al. (2015) [19] for piglets having in diet 4% OMWW. By contrast, in the study of Gessner et al. (2013), piglets fed a lower concentration of polyphenols from 1% grape seed and grape marc extract diet, the liver TBARS levels did not differ between the two groups [6]. Gessner et al. [48] have also shown that supplementation of the diet with 1% of grape seed and grape marc meal extract does not exert antioxidative effects in the liver of healthy piglets. These different conclusions may arise from the fact that Gessner at al. [48] used in their study an extract of grape seed and grape marc and not the grape pomace itself as in the case of Gerasopoulos et al. [19] and this study. 

In kidneys, TBARS level decreased, a result also reported by Kafantaris et al (2018) in piglets fed 9% GP diet [18] and also by Gerasopoulos et al. (2015), in piglets fed 4% OMWW [19].

## 5. Conclusions

Aiming to assess the influence of a diet rich in polyphenols given to weaned piglets to improve their antioxidant status, this feeding trial showed that 5% dietary GP had no significant effect on the average daily gain and feed:gain, but significantly increased the average daily feed intake. The most important effect of the GP+ diet was the significant increase of the antioxidant activity in the liver, spleen and kidneys, key organs in animal physiology, and the good health state of the animals after weaning. The GP included in piglet feed showed antioxidant activity. The polyphenols from the feed were found to pass in the plasma, being thereafter metabolized in organs, exerting here an important and significant antioxidant activity.

## Figures and Tables

**Table 1:** Compound feed and calculated the nutrient content of experimental diets (%) [20].

Ingredients (%)	Control Diet	GP+ Diet
Corn	57.32	53.90
Rice meal	-	-
Wheat	10.00	8.00
Sunflower meal (31.94% CP)	5.00	5.00
Soybean meal (44% CP)	16.00	16.00
Sunflower oil	0.20	0.70
Milk powder	3.00	3.00
Corn gluten meal (60% CP)	4.00	4.00
Grape pomace	-	5.00
Monocalcium phosphate	1.25	1.25
Limestone	1.56	1.48
NaCl	0.20	0.20
DL-Methionine	0.03	0.05
L-Lysine HCL	0.34	0.32
Choline	0.10	0.10
Mineral–vitamin premix	1.00	1.00
Calculated nutrient content according to [21]		
Dry matter (%)	88.31	88.01
Crude Protein (%)	18.29	18.36
Digestible crude protein (%)	14.98	15.06
Fat (%)	2.85	2.96
Crude fiber (%)	4.07	5.30
Metabolizable energy (Kcal/kg)	3169	3147
Lysine (%)	1.08	1.08
Digestible Lysine (%)	0.92	0.92
Met + Cys (%)	0.65	0.65
Calcium (%)	0.90	0.90
Phosphorus (%)	0.65	0.65

**Table 2:** Total polyphenols content and antiradical (antioxidant) activity (AAR) of the compound feeds and of their ingredients.

**Sample**	**Acetone Extraction**		**Methanol Extraction**	
	**Total Polyphenols** (mg GAE/100g raw material)	**AAR (DPPH Assay)** (μM Trolox equivalents)	**Total Polyphenols** (mg GAE/100g raw material)	**AAR (DPPH Assay)** (μM Trolox Equivalents)
**Ingredients**				
Corn Grains	694.48 ± 3.02 ^g,†^	237.39 ± 6.62 ^g,†^	682.24 ± 6.42 ^f,†^	253.68 ± 6.98 ^f,‡^
Wheat Grains	697.39 ± 11.64 ^f,g,†^	297.74 ± 3.99 ^f,†^	719.1 ± 6.89 ^e,‡^	273.92 ± 4.38 ^e,f,‡^
Sunflower Meal	1588.55 ± 69.96 ^a,†^	683.10 ± 4.37 ^a,†^	942.5 ± 27.23 ^a,‡^	678.85 ± 4.87 ^a,†^
Soybean Meal	839.07 ± 14.78 ^c,d,e,†^	644.64 ± 9.79 ^b,†^	746.14 ± 6.1 ^d,e,‡^	466.8 9 ± 7.79 ^c,‡^
Dried Grape Pomace	853.32 ± 19.75 ^b,c,d,†^	585.57 ± 3.61 ^c,†^	781.37 ± 7.26 ^b,‡^	482.99 ± 4.87 ^b,‡^
**Compound feeds**				
Control (GP−)	743.97 ± 10.66 ^e,f,g,†^	417.51 ± 2.46 ^e,†^	677.22 ± 7.44 ^g,‡^	233.78 ± 10.25 ^g,‡^
Grape Pomace (GP+)	810.60 ± 26.40 ^d,e,†^	454.36 ± 12.88 ^d,†^	749.95 ± 2.22 ^c,d,†^	410.52 ± 10.46 ^d,‡^

GP− = control group fed diet without grape pomace, GP+ = experimental group fed with grape pomace diet, GAE = gallic acid equivalents. Values represent the mean of triplicate determinations (n = 3) ± SEM. ^a,b,c,d,e,f,g^ Values within a column with different letter are statistically significant at *p* < 0.05. ^†,‡^ Values within a row with a different symbol are statistically significant at *p* < 0.05

**Table 3:** Effects of grape pomace (GP) diet on selected blood biochemical parameters *.

Items	15 Days				36 Days				*p*-Value		
	GP−	SEM	GP+	SEM	GP−	SEM	GP+	SEM	Diet	Time	D × T
Glucose (mg/dL)	96.81	4.58	100.05	3.73	90.24	1.73	80.76	3.40	0.826	0.09	0.335
Total Cholesterol (mg/dL)	83.32	3.49	76.89	2.18	83.30	1.43	85.65	1.67	0.388	0.069	0.068
Triglycerides (mg/dL)	65.91^a^	0.76	63.48^a^	2.90	62.95^a^	3.85	48.18^b^	3.46	0.07	0.040	0.046
Phosphorus (mg/dL)	7.77^c^	0.34	7.82 ^b,c^	0.21	8.62 ^a,b^	0.12	8.69^a^	0.13	0.778	0.000	0.982
Calcium (mg/dL)	9.52 ^a,b^	0.24	10.16^a^	0.27	8.97^b^	0.33	9.20 ^a,b^	0.30	0.141	0.012	0.479
Iron (ug/dl)	134.77	9.53	147.25	12.19	130.24	8.63	127.39	2.99	0.595	0.183	0.399
Magnesium (mg/dL)	1.36	0.05	1.67	0.18	1.49	0.06	1.46	0.03	0.155	0.703	0.103
Total protein (mg/dL)	5.13	0.19	5.23	0.12	5.11	0.19	5.24	0.17	0.515	0.963	0.948
Albumin (g/L)	2.99^c^	0.10	3.39^b^	0.09	3.97^a^	0.02	3.99^a^	0.01	0.004	0.000	0.008
Bilirubin (mg/dL)	0.12	0.013	0.11	0.010	0.13	0.015	0.12	0.013	0.451	0.451	1.000
Urea (mg/dL)	20.03	1.34	17.38	1.33	21.08	1.3	19.19	1.65	0.117	0.319	0.790
Creatinine (mg/dL)	0.94	0.03	0.98	0.035	0.94	0.04	1.02	0.04	0.123	0.576	0.672
ALP (U/L)	127.30	5.97	155.50	9.92	138.04	8.36	140.48	5.13	0.779	0.051	0.098
ALT (U/L)	50.43	2.43	53.92	4.89	52.31	2.53	56.56	4.13	0.296	0.540	0.918
AST (U/L)	49.17	4.02	45.64	3.36	47.33	2.70	50.06	3.05	0.976	0.843	0.354
GGT (U/L)	25.23	1.79	31.16	3.69	26.12	2.21	26.88	2.46	0.213	0.524	0.333

* GP−= group fed with control diet, GP+ = group fed with 5% dried grape pomace diet. Values represent the mean of n = 10 determinations ± SEM. ^a,b,c^ Means that do not share the same letter within a raw are significantly different.

**Table 4:** Total polyphenols content in blood and its fractions (mg GAE /L sample).

Items	After 15 Days				After 36 Days				*p*-Value		
	GP−	SEM	GP+	SEM	GP−	SEM	GP+	SEM	Diet (D)	Time (T)	D × T
plasma	592.62^b^	2.30	592.90^b^	5.30	594.81^b^	3.74	615.52^a^	5.30	0.022	0.008	0.026
cell sediment	600.49	3.65	614.41	3.41	602.93	10.24	614.70	9.43	0.084	0.852	0.882
blood	635.28	8.10	643.57	2.94	649.37	3.70	657.46	7.01	0.169	0.022	0.987

**Table 5:** The total polyphenols level (mg GAE/100g raw material) and antioxidant activity-DPPH (AAR, Trolox Equivalents—TE) in organs (liver, spleen, kidney, mesenteric lymph nodes, heart and brain) and Longissimus dorsi muscle at the end of the trial.

**Items**	**Total Polyphenols** (mgGAE/100g raw material)			**Antioxidant Activity** (AAR by DPPH assay) (μM Trolox equivalents)		
	**GP−**	**GP+**	***p*-Value**	**GP−**	**GP+**	***p*-Value**
**Organs**						
Liver	175.04 ± 1.39	171.99 ± 1.24	0.119	109.64 ± 9.86 ^b^	144.41 ± 7.02 ^a^	0.009
Spleen	155.32 ± 3.85	147.56 ± 1.68	0.072	99.87 ± 6.88	98.48 ± 9.53	0.909
Kidney	143.74 ± 0.59 ^b^	147.80 ± 1.26 ^a^	0.019	35.38 ± 6.57	41.90 ± 6.80	0.506
Mesenteric lymph nodes	141.69 ± 1.05	140.40 ± 1.37	0.464	110.36 ± 7.80	112.36 ± 10.96	0.886
Heart	140.10 ± 1.26	140.35 ± 1.08	0.882	8.51 ± 2.13	11.27 ± 2.80	0.474
Brain	153.28 ± 1.81	150.90 ± 2.00	0.394	63.29 ± 8.65	59.75 ± 9.12	0.782
**Muscle**						
L. dorsi muscle	122.93 ± 1.18	124.71 ± 1.03	0.270	21.53 ± 3.40	21.17 ± 2.66	0.935

GP−= group fed with control diet, GP+ = group fed with 5% dried grape pomace diet. Values represent the mean of n = 10 determinations ± SEM. ^a,b^ Means that do not share the same letter within a raw are significantly different.

**Table 6:** UV-Vis Absorption maxima for the of the overlaid spectra subtractions of liver, spleen and kidney samples of piglets fed with a control or 5% GP diet.

Samples	λ max (nm)		
Liver	-	328	430
Spleen	286.5	344	415
Kidney	284.6-289	336	443
